# Evaluation of levels of uric acid and lipid profile in hospitalized patients with diabetes

**DOI:** 10.1186/s13104-023-06429-5

**Published:** 2023-07-24

**Authors:** Haniyeh Sadat Fayazi, Seyyedeh Sahereh Mortazavi Khatibani, Behrang Motamed, Maryam Yaseri

**Affiliations:** Department of Internal Medicine, School of Medicine, Razi Hospital, Guilan University of Medical Sciences, Rasht, Iran

**Keywords:** Triglyceride, Serum uric acid, Lipids, Diabetics, Lipid profile

## Abstract

**Objective:**

Diabetes is the most common metabolic disorder that leads to various complications, and among these complications, disruption in the lipid profile and serum uric acid (SUA) is one of the significant cases that can lead to the deterioration of the health status of patients with diabetes. So, we aimed to evaluate the level of SUA and lipid profiles in patients with diabetes. A total of 230 patients with diabetes who were admitted to Razi Hospital, Rasht, Iran, were enrolled in this study. Demographical data and clinical characteristics of the patients include gender, body mass index (BMI), duration of diabetes, history of smoking, FBS, HbA1c, SUA, Creatinine (Cr), Cholesterol (Chol), low-density lipoprotein (LDL), high-density lipoprotein (HDL), triglycerides (TG), retinopathy, hypertension, ischemic heart disease (IHD), and microalbuminuria were recorded. All data were analyzed using the SPSS version 21 by a significant level < 0.05.

**Result:**

According to our results, 70 were male, and 160 were female, with a mean age of 57.36 ± 8.05 years and a mean BMI of 28.10 ± 4.62. The most frequent comorbidities were hypertension, 67%. The serum level of FBS, HBA1c, SUA, Cr, Chol, LDL, HDL, and TG were 191.47 ± 71.66 mg/dL, 7.94 ± 1.21 mg/dL, 5.65 ± 1.95 mg/dL, 0.94 ± 0.16 mg/dL, 167.28 ± 45.22 mg/dL, 95.91 ± 37.03 mg/dL, 39.78 ± 10.44 mg/dL, and 186.75 ± 76.65 mg/dL, respectively. Only UA had a significant relationship with TG level (*P* < 0.05).

## Introduction

Uric acid (UA) is a final enzymatic product in humans’ degradation of purine nucleosides and free bases. The pathway of purine catabolism in humans is shorter than in other primates. Consequently, humans have to deal with higher levels of UA in the blood and are susceptible to hyperuricemia and gout [1, 2]. High serum UA (SUA) levels, and hyperuricemia, are associated with gout, hypertension, kidney stones, kidney disease, cardiovascular complication, and metabolic syndrome [3]. Some diseases, such as metabolic syndrome and type 2 diabetes mellitus (T2DM), are often considered lifestyle-related diseases due to their pathological basis [4–6]. Diabetes has become an epidemic over the past few decades as one of the most significant global health emergencies of the 21st century. According to a report by the International Diabetes Federation, diabetes mellitus prevalence is likely to increase to 642 million by 2040 [7]. Despite the high prevalence of diagnosed diabetes, almost half of diabetic individuals are unaware of their disease. Diabetes is contributed to a higher rate of morbidity and mortality in the future half of them occur due to cardiovascular complications [8]. Pieces of evidence have shown that an abnormal lipid profile, along with T2D, has a close relationship with insulin resistance, which is the major component of other metabolic disorders [9, 10]. Insulin resistance is associated with a high level of very-low-density lipoprotein (VLDL), high concentrations of serum triglycerides (TG), and serum high-density lipoprotein (HDL).

Consequently, the lipid profile is highlighted in nearly all follow-up plans of T2DM and assists as a severe risk factor [11, 12]. Dyslipidemia is frequently associated with obesity, T2DM, and metabolic syndrome. Dyslipidemia includes elevated TG, normal or slightly elevated low-density lipoprotein cholesterol (LDL), and decreased levels of HDL [13]. By changing the worldwide lifestyle, the prevalence of dyslipidemia has grown, especially in developing countries [14]. Elevated SUA levels or hyperuricemia are significantly associated with dyslipidemia [15].

By considering gender differences in some complications and diseases, it is still not clear whether the relationship between SUA levels and dyslipidemia bares consistent across different genders, which merits consideration. A study reported a strong positive association between SUA levels and dyslipidemia in the male gender but not in females [16]. Only a few studies were conducted on the association between lipid profile and SUA in patients with diabetes, who are usually engaged with those two dysregulations in Iran. Early prevention of hyperuricemia and dyslipidemia may help reduce further complications in patients with diabetes. In this regard, we aimed to conduct this study to evaluate the level of SUA and dyslipidemia in patients with diabetes in North Iran and evaluate the demographical and clinical characteristics.

## Main text

### Patients and methods

#### Study design, categorization of patients, and sample size

The data of this cross-sectional study were collected from 230 adult patients with diabetes, selected by census method, and referred to Razi Hospital, Rasht, Iran, from March 2019 to March 2020. Diabetes in these patients was confirmed by specialists with a level of hemoglobin A1c (HbA1c) > 6.5% and fasting blood sugar (FBS) > 126 mg/dl [17] through laboratory results or who were on blood glucose control medication. Individuals with a history of kidney disease, pregnant ones, patients who have recently undergone surgery, and patients with incomplete information were excluded from the study. Before the study, the informed consent form was fulfilled by patients. The study design was approved by the ethical committee of Guilan University of Medical Sciences (IR.GUMS.REC.1399.171).

Demographical data and clinical characteristics were collected from patients’ medical records in the hospital archive. They included gender, gender, body mass index (BMI), duration of diabetes, history of smoking, retinopathy, hypertension, ischemic heart disease (IHD), microalbuminuria, and also laboratory findings that included HbA1c, FBS, UA, Creatinine (Cr), Cholesterol (Chol), LDL, HDL, and TG, which were analyzed by auto-analyzer (BS-800, Mindray, UK, Ltd). The cut-off values for hypercholesterolemia (≥ 240 mg/dL), hypertriglyceridemia (≥ 200 mg/dL), and low HDL-cholesterolemia (≤ 40 mg/dL).

#### Statistical analysis

Categorical variables are frequency and percentage, while continuous variables are Mean ± S.D. The chi-square test or exact Fisher test was applied to evaluate the association between two categorical variables. Statistical calculations were performed using IBM SPSS Statistics for Windows, version 21, with a statistically significant level of less than 0.05.

## Results

According to our result, among 230 participants, 70 were male, and 160 were female, with a mean age of 57.36 ± 8.05 years and a mean BMI of 28.10 ± 4.62. The average duration of diabetes existence was 11.68 ± 7.13 years. The level of FBS, HBA1c, UA, Cr, Chol, LDL, HDL, and TG were 191.47 ± 71.66 mg/dL, 7.94 ± 1.21 mg/dL, 5.65 ± 1.95 mg/dL, 0.94 ± 0.16 mg/dL, 167.28 ± 45.22 mg/dL, 95.91 ± 37.03 mg/dL, 39.78 ± 10.44 mg/dL, and 186.75 ± 76.65 mg/dL, respectively. The most frequent comorbidity in the patients was hypertension 67% (154 n) (Table 1).

**Table 1:** Frequency of demographical and clinical data of patients

Variables		Number	Percentage
**Gender**	Male	70	30.4%
	Female	160	69.6%
**History of smoking**	No	187	81.3%
	Yes	43	18.7%
**Retinopathy**	No	139	60.4%
	Yes	91	39.6%
**Hypertension**	No	76	33%
	Yes	154	67%
**Ischemic heart disease**	No	173	75.2%
	Yes	57	24.8%
**Microalbuminuria**	No	124	53.9%
	Yes	106	46.1%
**Mean ± SD**			
**Age**	57.36 ± 8.05		
**BMI**	28.10 ± 4.62		
**Duration of diabetes**	11.68 ± 7.13		
**FBS**	191.47 ± 71.66		
**HBA1C**	7.94 ± 1.21		
**Uric Acid**	5.65 ± 1.95		
**Creatinine**	0.94 ± 0.16		
**Cholesterol**	167.28 ± 45.22		
**LDL**	95.91 ± 37.03		
**HDL**	39.78 ± 10.44		
**Triglyceride**	186.75 ± 76.65		

Low-density lipoprotein (LDL); High-density lipoprotein (HDL).

Qualitative variables are described as frequency and percentage, while continuous variables are as Mean ± S.D

Due to Table 2, only UA had a statistically significant relationship with TG (*P* = 0.03), so 55% of patients with SUA level under 6.8 mg/dL represented 150 mg/dL ≤ TG, and 77% of patients with SUA level above 6.8 mg/dL, had 150 mg/dL < TG. Only 5.3% of patients with SUA levels under 6.8 mg/dL and 8.2% with SUA levels above 6.8 mg/dL had 240 mg/dL ≤ Chol level. Also, among patients with SUA levels under 6.8 mg/dL, 6.5% had 160 mg/dL ≤ LDL and 83.4% had HDL < 40 mg/dL (in males) and HDL < 50 mg/dL (in females). In the group of patients with SUA levels above 6.8 mg/dL, about 4.9% had 160 mg/dL ≤ LDL and 78.7% had HDL < 40 mg/dL (in males), and HDL < 50 mg/dL (in females).

**Table 2:** Comparison of the frequency of hyperlipidemia in patients with normal and abnormal Uric acid level

		Uric acid n(%)		Total	*P-*Value^*^
		< 6.8(mg/dL)	6.8(mg/dL)<		
**Triglyceride**	< 150 mg/dL	76 (45%)	14 (23%)	90 (39.1%)	0.003
	150 mg/dL ≥	93 (55%)	47 (77%)	140 (60.9%)	
**Cholesterol**	< 200 mg/dL	130 (76.9%)	44 (72.1%)	174 (75.6%)	0.659
	200 mg/dL ≥ Chol < 240 mg/dL	30 (17.8%)	12 (19.7%)	42 (18.3%)	
	240 mg/dL ≥	9 (5.3%)	5 (8.2%)	14 (6.1%)	
**LDL**	< 100 mg/dL	99 (58.6%)	38 (62.3%)	137 (59.6%)	0.840
	100 mg/dL ≥ LDL < 160 mg/dL	59 (34.9%)	20 (32.8%)	79 (34.3%)	
	160 mg/dL ≥	11 (6.5%)	3 (4.9%)	14 (6.1%)	
**HDL**	< 40 mg/dL in male < 50 mg/dL in female	141 (83.4%)	48 (78.7%)	189 (82.2%)	0.407
	40 mg/dL < HDL < 100 mg/dL in male 50 mg/dL < HDL < 110 mg/dL in female	28 (16.6%)	13 (21.3%)	41 (17.8)	
**Total**		169 (73.5%)	61 (26.5%)	-	

Low-density lipoprotein (LDL); High-density lipoprotein (HDL).

^*^Chi-square test or exact Fisher test were applied to evaluate the association between two categorical variables. The statistical significance was evaluated at the level of 0.05

## Discussion

The results of the present study can be summarized as follows; First, hypertension was the most frequent comorbid in patients with diabetes. Secondly, abnormal SUA was associated with a significantly higher TG level. In addition, abnormal microalbuminuria was present in almost half of T2DM patients. Patients with diabetes are more susceptible to developing dyslipidemia (quantitative and qualitative), with a reported prevalence of 24–40% [18–20].

IN THEIR STUDY, Kodama S et al. reported significant hyperuricemia in patients with T2DM. They also suggested that UA had a crucial role in worsening insulin resistance by inhibiting the bioavailability of nitric oxide, which is essential for insulin-stimulated glucose uptake [21]. Another study represented that high UA levels are significantly associated with prediabetes, indicating that UA has a significant role in glucose metabolism. Also, they reported a significant positive association between dyslipidemia and serum UA [22].

Some studies compare SUA levels with lipid profiles in patients with diabetes; as reported in a study by Mohammedsaeed, dyslipidemia (high level of LDL, TG, and low level of HDL) and HbA1c were highly correlated with UA in patients with diabetes [23]. Moreover, Agrawal et al. reported a significantly lower mean of HDL in patients with diabetes, with and without retinopathy, than in healthy individuals. Mean HbA1c and mean FBS significantly correlated with UA (*P* = 0.01 and *P* = 0.02, respectively) in both groups. A deranged HDL profile and a significant correlation between glycemic control and HbA1c and UA were thus found in two study groups compared to the healthy control group [24].

Due to our data, retinopathy was reported in 39.6% of the study population. The association of dyslipidemia with diabetic retinopathy is also a subject of considerable debate. In many studies.

Dyslipidemia has not been reported to be associated with diabetic retinopathy [25–27]. Although, some other studies have illustrated a significant association between hypercholesterolemia and LDL with the severity of retinal hard exudates [28, 29]. Also, as demonstrated in our study, a higher frequency of hypertension was the most comorbidities among the study population. In line with our results, in some other studies, it has been reported that dyslipidemia is associated with an increased risk of hypertension [30, 31]. Gaubert et al. illustrated the important role of high SUA levels in hypertension-associated morbidities and should be considered in patients with chronic hyperuricemia [32]. Moreover, about 24.8% of patients with diabetes had a history of IHD. As reported in some studies, dyslipidemia and hyperglycemia can play a key role in cardiovascular dysfunction in at-risk adolescents with diabetes [33, 34].

Microalbuminuria was reported at 46.1% in patients with diabetes due to our results. According to the result of a study, hypoalbuminemia rates in the non-survivors and survivor groups were 76.7% and 57.8%, respectively (*P* = 0.019). Hyperuricemia rates were 74.4% and 57.1%, respectively (*P* = 0.033) [35]. In patients with T2DM, it was reported that SUA levels correlated with urinary albumin excretion, which was associated with microalbuminuria in these patients [36, 37]. In T2DM patients with abnormal albuminuria, the lipid profile was characterized by lower Chol levels and a tendency toward higher TG levels than those with normal albuminuria. The lower level of HDL was the most frequent of isolated dyslipidemia, and about 70% of patients with abnormal albuminuria had atherogenic dyslipidemia [38].

High lipid profile, except the LDL, was reported in the group with 6.8 mg/dL < SUA in comparison to the group with SUA < 6.8 mg/dL, in which this elevation has represented no statistically significant differences (*P* > 0.05). As reported in Liu et al.‘ study, the proportion of individuals with abnormal levels was higher in the hyperuricemia group than in the normal group [39]. A study by Vekic et al. reported that the highest SUA was associated with significantly smaller sizes of LDL and HDL [40]. In our study, comparing LDL levels among the two groups illustrated the same result for LDL, although it represented no statistically significant differences. Several authors concluded elevated SUA reflects insulin resistance [41, 42]. However, it is controversial whether hyperuricemia plays a fundamental role in diabetes. Lu et al. confirmed no causal association between hyperuricemia and diabetes [43]. Elevated SUA accelerates but does not cause diabetes because SUA itself is insufficient to induce diabetes, although it can damage glucose tolerance and lead to insulin resistance [43].

## Conclusion

According to our results, a higher level of SUA was reported among diabetic patients with a higher level of TG. Also, conducting the follow-up cohort study can be helpful to achieve more robust results to help physicians monitor patients with diabetes to make an early diagnosis of either Elevated SUA or dyslipidemia, which can be helpful to prevent other adverse consequences.

## Limitations

The limitation limited access to the history of patients’ underlying disease and incomplete data on individuals’ diets and lifestyles were the current study’s limitations.

## Data Availability

The datasets used and analyzed during the current study are available from the corresponding author on reasonable request.

## Abbreviations

SUA: Serum uric acid
BMI: Body mass index
Cr: Creatinine
IHD: Ischemic heart disease
VLDL: Very-low-density lipoprotein
LDL: Cholesterol (Chol); low-density lipoprotein
HDL: High-density lipoprotein
TG: Triglycerides
IHD: Ischemic heart disease
HbA1c: Hemoglobin A1c
FBS: Fasting blood sugar
T2DM: Type 2 diabetes mellitus
